# The Story of the Insane from Year to Year

**Published:** 1900-11-24

**Authors:** 


### THE STORY OF THE INSANE FROM YEAR TO YEAR.

(Thirteenth Series.)

### CHESHIRE COUNTY ASYLUM AT UPTON.

On the last day of the year 1899 this Asylum contained 893 patients. There were 158 first admissions, 44 re-admissions, 86 recoveries, and 105 deaths. Calculated on the year's admissions the recoveries stand at 42·57 per cent., and calculated on the average number resident, the deaths stand at 12·33 per cent., both of these being considerably above the average in kindred institutions. Of the deaths, 45 were caused by cerebral and spinal diseases; 8 by pneumonia; 27 by phthisis; 3 by pleurisy, and 1 by enteritis. It would tend greatly to the usefulness of this table if the diseases were arranged in groups as is now almost universal in asylums. Of the year's admissions, 29 owed their insanity to moral causes; 30 to intemperance in drink; 44 to hereditary tendency, and 87 to previous attacks. In his report, Dr. Lawrence notes the high mortality due to consumption; but he offers no explanation of it, though he states that the mortality has not been affected by the means taken to prevent. These included the slaughter of tuberculous cows, as far as possible separating the diseased from the healthy and burning the sputa. Dr. MacDowall's remarks on the cross ventilation of the dormitories occur to us, and as a preventive measure it is undoubtedly useful. Four members of the subordinate staff received pensions during the year, and eighteen nurses and six attendants have obtained the St. John's Ambulance certificates. A class has been formed for further instructions in nursing. "The committee again desire to record their satisfaction with the manner in which the Asylum has been managed by Dr. Lawrence and the staff generally." The weekly charge to the Unions is 8s. 2d. per head.

### EARLSWOOD ASYLUM FOR IDIOTS AT REDHILL.

On January 1st, 1899, this asylum contained 362 patients, and by the end of the year the numbers had fallen to 554. There were 51 first admissions, 6 re-admissions, 45 discharged relieved, and 20 deaths. Of the year's admissions 15 are put down as caused by heredity, 1 to consanguineous marriage, 17 to trouble of the mother during pregnancy, and 9 to convulsions. Half of the deaths were caused by consumption, and 2 by pneumonia. It is undeniable that there is much in a name, and Dr. Caldecott proposes that Earlswood should be rechristened and henceforth be known as the "Training College for the Feeble-minded." He would further add a block to the asylum (or college) wherein he would place the helpless, uneducible, and the worst of the epileptics. There are three classes of patients in the asylum:—Free election cases, election cases with payment, and paying patients. The average cost per head of all classes is 19s. a week—say £50 a year; but the lowest sum at which private patients are admitted is £68 5s. At many of our County Asylums private patients are received at sums varying from 15s. to 20s. a week, and there is no reason why Earlswood should not receive suitable cases at a small addition to the actual cost.

